# Ring artifacts correction in compressed sensing tomographic reconstruction

**DOI:** 10.1107/S1600577515010176

**Published:** 2015-07-14

**Authors:** Pierre Paleo, Alessandro Mirone

**Affiliations:** a ESRF, 71 Avenue des Martyrs, 38000 Grenoble, France; b Université de Grenoble, Gipsa-Lab, 11 Rue des Mathématiques, 38400 Saint-Martin-d’Hères, France

**Keywords:** tomography, artifacts, compressed sensing

## Abstract

Ring artifacts are a very common problem in tomographic reconstruction, and numerous methods exist to either pre-process the sinogram or correct the reconstructed slice. A novel approach to perform the correction as part of the reconstruction process is presented. It is shown that for iterative techniques, which amount to optimizing an objective function, the ring artifacts correction can be easily integrated in the formalism, enabling simultaneous slice reconstruction and ring artifacts correction. This method is tested and compared with mainstream correction techniques for both simulated and experimental data. Results show that the correction is efficient, especially for undersampled datasets. This technique is included in the *PyHST2* code which is used at the European Synchrotron Radiation Facility for tomographic reconstruction.

## Abstract 2

The formalism of iterative tomographic reconstruction with sparsity inducing penalty is extended to enable ring artifacts correction.

## 1. Introduction   

### 1.1. Rings artifacts in tomographic reconstruction   

During a tomographic acquisition process, some flaws in the experimental setup can lead to unwanted artifacts appearing on the reconstructed slice. Ring-shaped features are a well known example of such artifacts. Even after pre-processing steps like flat-field correction and median filtering, these artifacts can remain and are detrimental to the reconstruction quality. Therefore, multiple techniques have been developed to tackle this problem.

Generally speaking, ring artifacts have various possible causes. The presence of defective pixels in the detector leads to sharp artifacts, while dust on the scintillator crystal can form large artifacts. Experimental flaws can also include vibration of the monochromator or tilt of the rotation axis. In almost all cases, the defects appear as lines in the sinogram since they are independent of the projection angle. These spurious lines give rise to ring-shaped artifacts in the reconstructed object.

### 1.2. Related work   

Various techniques have been proposed in the literature to reduce or suppress the rings artifacts. As reported by Rashid *et al.* (2012 ▸), these techniques can be classified into two groups: sinogram pre-processing and reconstructed images post-processing. The pre-processing methods aim at detecting and correcting the spurious lines in the sinogram before applying the reconstruction process, thus, rings do not form if the method succeeds. A recent work (Miqueles & Bermúdez, 2014 ▸) reports a compressed sensing approach for rings artifacts reduction using a Total Variation denoising of the sinogram before calling the reconstruction routine. It is a generalization of Titarenko’s algorithm (Titarenko *et al.*, 2010 ▸) which consists of a regularization of the sinogram. This can also be classified in the sinogram pre-processing techniques.

On the other hand, post-processing techniques work directly on the reconstructed image, trying to extract the concentric circles and filter them. These methods often perform a transformation into polar coordinates to transform the concentric circles into straight lines (Prell *et al.*, 2009 ▸).

A comprehensive comparison of ring artifact removal methods can be found by Rashid *et al.* (2012 ▸). Although these methods certainly provide satisfactory results in their limited framework, the authors report that no existing method is really suitable for correcting different types of rings, since they always introduce other distortions. Thus, the ring removal problem cannot be considered as solved and is subject to continual efforts. In this paper, a new approach to correct the ring artifacts in a compressed-sensing framework is presented. In this technique, the correction is intrinsically part of the reconstruction process, hence can be neither viewed as sinogram pre-processing nor slice post-processing. The basic idea is to split the sinogram into two components, one containing the genuine sinogram and the other containing the artifacts component. This approach bears some similarities with a recent work (Mohan *et al.*, 2014 ▸) where the artifacts model is also included in the objective function which is optimized in a non-homogeneous iterative coordinate descent. However, the aforementioned method uses a L2 minimization, while compressed sensing methods typically use Total Variation or L1 regularization, which is adapted for undersampled data.

## 2. Preamble   

In this section, we introduce the principle and the formalism of compressed sensing tomographic reconstruction. This formalism is extended in §3 for ring artifacts correction.

### 2.1. Compressed sensing tomographic reconstruction   

Computed tomography aims at reconstructing an image 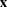 from a set of projections 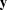 = 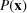. Here 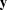 denotes the acquired sinogram, 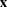 is the slice to be reconstructed and *P* is the projection operator (while its adjoint 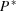 = 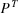 is the back-projection operator). The classical filtered back-projection algorithm enables the image to be reconstructed, but the number of projections should be of the same order of the number of rows in the image to have an acceptable reconstruction according to the Shannon–Nyquist sampling theorem. This is often impracticable, and the subsampling leads to artifacts in the reconstructed image.

Compressed sensing techniques exploit *a priori* knowledge on the image, like its sparsity, in order to bypass this limitation. Instead of computing a closed form solution like in the filtered back-projection technique, tomographic reconstruction by compressed sensing amounts to an optimization problem,
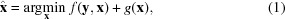
where 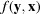 is a fidelity term of 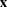 with respect to the acquired data 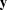 [henceforth 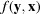 is denoted 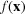], and 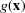 contains *a priori* knowledge on the image. In general, the regularization term 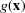 makes the problem non-smooth, which precludes from using usual gradient-like algorithms 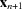 = 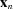 − 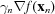.

Advances in convex analysis provide adapted methods, based on proximal splitting methods (Combettes & Pesquet, 2009 ▸), which are a generalization of projected gradient. One instance is the Iterative Shrinkage-Thresholding Scheme (ISTA; see, for example, Daubechies *et al.*, 2004 ▸). For a functional split into a smooth term *f* and a non-smooth term *g*, the first-order condition at an optimum 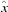 reads
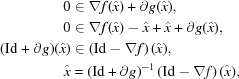
which suggests the use of a fixed point iterative scheme. Here 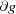 is the subgradient of *g*, and the operator 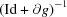 is called the *proximal operator*,
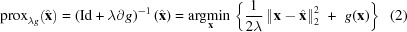
so that one step of ISTA reads
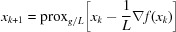
where *L* is the Lipschitz constant of the gradient 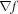 of the fidelity term. Usually, the data fidelity term is an L2 norm, so the calculation of the proximity operator of *f* is straightforward. On the other hand, the proximity operator 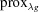 of the regularization term does not always have a closed form expression.

### 2.2. Total variation regularization   

Depending on the regularization term used in (1), the reconstruction can yield very different results. If the regularization term is null, the problems amount to a least-squares reconstruction. This approach tends to blur the edges in the reconstructed slice. A commonly used regularization term for image denoising is the Total Variation which was introduced by Rudin *et al.* (1992 ▸) as the Rudin–Osher–Fatemi model. This prior has the property to preserve the image edges, which is essential especially in tomographic reconstruction where the object in the sample should be distinguishable. In a discrete framework, the (isotropic) Total Variation (TV) is the L1 norm of the image gradient magnitude: 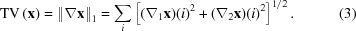
Given an observed sinogram 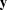, the Total Variation tomography reconstruction problem aims at finding the regularized image 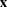 satisfying
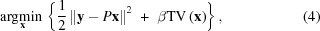
where β weights the regularization with respect to the data fidelity term, and *P* is the projection matrix. If *P* is equal to the identity matrix, the problem (4) is equivalent to the so-called Total Variation denoising problem: 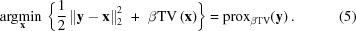
Knowing the proximal map of the Total Variation regularization term (see, for example, Michel *et al.*, 2011 ▸), the denoising problem can be solved by an iterative shrinkage-thresholding scheme. Accelerated versions of ISTA, like the Nesterov algorithm (Nesterov, 2007 ▸) or FISTA (Beck & Teboulle, 2009 ▸), are particularly efficient for solving this problem.

However, tomographic reconstruction is not a simple denoising problem, since a projection matrix *P* appears in the problem (4). Constructing a smooth dual of (4) would entail inverting 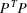 which is an ill-posed problem. The Total Variation tomographic reconstruction can be tackled with two nested FISTA loops, each iteration being the solution of a denoising subproblem (Beck & Teboulle, 2009 ▸),
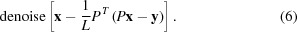
Here *L* is the Lipschitz constant of 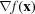 = 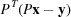 which is calculated using the power method.

### 2.3. Dictionary Learning   

Total Variation regularization performs well for piecewise-constant images since edges and uniform regions are re­inforced. However, for non-piecewise-constant images, the *cartoon* effect might be prejudicial for the reconstruction quality. Thus, another regularization technique has to be considered for such images.

Most natural images have an intrinsic sparsity which can be recovered by an adapted transform or by building the best sparsifying basis. The latter technique is called Dictionary Learning (DL). Given a set of *N* acquired signals 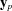, a number of 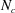 basis vectors (or atoms) 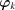 are built. Each signal 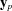 is expressed as a linear combination 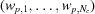 of the 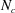 atoms. Dictionary Learning is a joint optimization of the atoms *D* and the coefficients 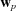 under the constraint of sparsity: 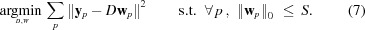
Here 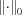 denotes the zero norm counting the number of non-zero components of a vector.

In image processing problems, the image 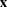 is divided into *N* square patches of size 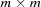 pixels. Every patch area of index 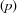 is expressed as a linear combination of the atoms 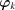: 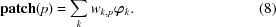
That is, for pixel 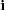 of the image 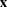: 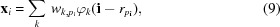
where 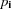 denotes the patch containing the pixel 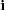, and 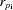 is the center of this patch so that 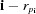 belongs to the patch support. In equation (9), the atoms φ_*k*_ are already known, which corresponds to a dictionary learned off-line.

Dictionary Learning techniques have been proposed for sparse representation in X-ray tomography reconstruction (Liao & Sapiro, 2008 ▸; Xu *et al.*, 2012 ▸). They turn out to be especially efficient in achieving a good reconstruction quality even when few projections are available. To prevent discontinuities at the patch borders, a functional enabling the patches to overlap is proposed in *PyHST2* (Mirone *et al.*, 2014 ▸). The tomographic reconstruction problem is
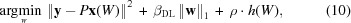
where 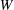 = 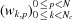 is the matrix containing the patches coefficients, and *h* is a term promoting coherence between the patches over the overlaps. The slice 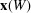 is a linear combination of the patches coefficients *W*, so the optimization is performed with respect to these coefficients.

In *PyHST2*, the optimization problem (10) is solved with the FISTA algorithm. The dictionary *D* is learned off-line with EK-SVD (Mazhar & Gader, 2008 ▸). It turned out that using the same dictionary for all the tests and the experimental data led to good reconstruction results, thus, the same dictionary was used for all the tests.

## 3. Rings correction in compressed sensing reconstruction   

We now present how rings correction can be handled directly in the reconstruction process by integrating additional variables in the functional to minimize. This approach is independent of the regularization used and can therefore be applied in various frameworks like Total Variation and Dictionary Learning. Sinogram pre-processing techniques modify the acquired data to filter the unwanted lines. This filtering often introduces new artifacts. On the other hand, image correction techniques can also add new artifacts when circular features are detected as artifacts; and the forward and backward Cartesian–polar coordinate transforms lead to a loss of precision even with a bilinear interpolation. When the rings correction is performed in the reconstruction process, the data are not modified.

**Figure 1:**
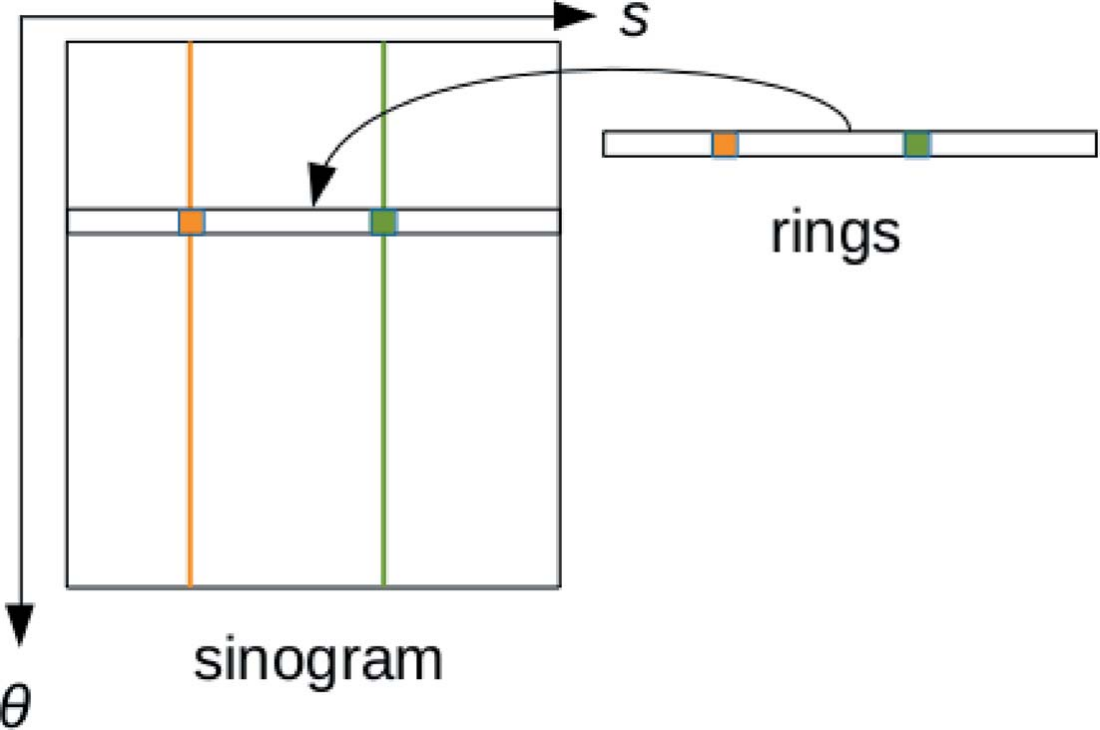
Principle of the rings separation. θ is the projection angle and *s* is the detector bin index. The vertical orange and green lines represent spurious lines giving rise to ring artifacts. The decomposition 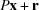 forces the ring values to be captured in the vector 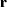 (independent of the projection angle). In the end, only the part without the rings 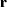 is returned.

In our approach, the rings correction consists of splitting the sinogram into two components: the ‘genuine’ sinogram and the spurious straight lines giving rings after back-projection. Although ring artifacts have various causes, they often appear in the sinogram as lines which are almost constant along the projection angle. Thus, a natural approach is to model the rings by constant lines in the sinogram. In iterative techniques, the rings correction can be handled by additional variables 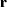 in the fidelity term 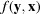: rings variables are stacked in a vector and added to the sinogram for each projection. The fidelity term for one projection reads
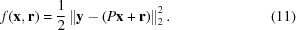
Here 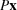 and 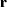 do not have the same dimensionality; 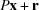 means that a vector of rings variables is added to each line of the sinogram as illustrated in Fig. 1 ▸.

A recent work (Mohan *et al.*, 2014 ▸) also implements this approach in a maximum *a posteriori* (MAP) framework. More specifically, equation (3) therein describes a similar decomposition of the sinogram 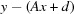 where *y* is the sinogram, *A* the projection operator, *x* the slice and *d* the rings vector (also constant along the projection angle).

We emphasize the fact that the sinogram decomposition into a genuine sinogram 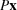 and spurious rings 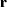 is not a pre-processing technique; the rings removal is intrinsically part of the reconstruction process. At each iteration, the image 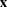 and the rings variables 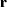 are adapted to minimize the energy 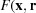).

This splitting is done in the reconstruction process, so the two components are updated after each iteration. In the end, only the valid sinogram component is kept while the rings variables are discarded. We give two examples of frameworks using this approach: Total Variation regularization and Dictionary Learning reconstruction.

### 3.1. Rings correction in Total Variation framework   

When the sparsity-inducing prior is the Total Variation, the functional 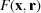 is
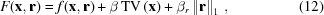
β being a parameter weighting the relative importance of spatial regularization, and 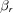 being a penalization parameter for the rings.

 While sinogram pre-processing techniques filter the lines parallel to the projection angle, this approach forces the sinogram to be decomposed as a sinogram 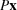 and rings variables 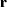. The sparsity constraint 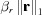 forces the rings variables to have only a few not null components, since the L1 norm is a good approximation of the sparsity-inducing L0 norm.

The minimum of 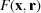 is found with the Total Variation regularization solver presented in §2.2. This iterative algorithm has a step size 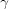 = 1/*L* where *L* is the Lipschitz constant of the gradient 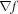.

This constant is an upper bound of the largest eigenvalue of the Hessian 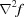. Since the gradient is now taken with respect to both image and rings variables, the Hessian is
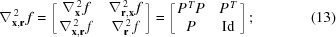
its largest eigenvalue is calculated using the power method.

Once the Lipschitz constant *L* is obtained, one iteration of the FISTA denoising algorithm needs to compute
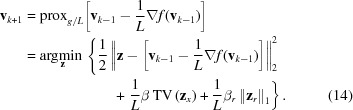
Here 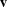 denotes the augmented vector 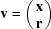
containing both image and rings variables, and 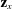 (respectively 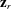) is the part of vector 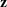 containing image (respectively rings) variables. Since the squared L2 norm is separable, the proximal operator (14) can be written
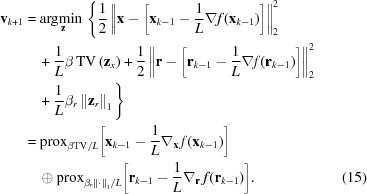
In short, the proximal operator is separable with respect to the image and rings variables. This is convenient because rings and image variables can be updated by solving two separate subproblems in a FISTA iteration.

The first term in (15) is the denoising problem (5). The second term is the proximity operator of the L1 norm, which has a closed-form expression called the *soft thresholding operator*, 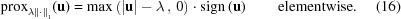



### 3.2. Rings correction in Dictionary Learning framework   

Similarly to Total Variation (§3.1), the proximal operator is separable with respect to patch variables *W* and rings variables 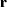. The problem (10) becomes
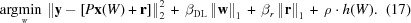
On the other hand, in this case, the non-smooth term is only the L1 norm which has a closed-form proximal operator (16). Thus, the denoising problem is straightforward, and only one FISTA loop is required.

### 3.3. Preconditioning the optimization problem   

For iterative techniques, a crucial parameter acting on the convergence rate is the condition number of the objective function. When possible, a preconditioner is used to fasten the convergence rate (Benzi, 2002 ▸). In both cases of Total Variation (§3.1) and Dictionary Learning (§3.2), the optimization process can be speeded up by a preconditioner. It is well known that in the back-projected slices the low spatial frequencies are overrepresented with respect to high frequencies. In our case, this leads to an ill-conditioned reconstruction problem. Therefore, a ramp filter is applied in the Fourier domain to give the proper weight to low and high frequencies.

The filtering is done before back-projection in every iteration, using a discretized version of the high-pass ramp filter (Murrell, 1996 ▸) which does not set to zero the zero frequency component. If there is only one iteration, the optimization then reduces to the standard filtered back-projection. This multiplication by a ramp filter can be seen as a preconditioner transforming an ellipsoidal (ill-posed) problem into a spherical problem, thus fastening the rate of convergence.

The fidelity term *f* then becomes
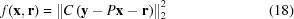
where *C* is the preconditioner. The gradient is
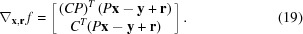
Here the operator 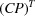 = 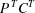 is the filtered back-projection; so the preconditioner is identified with the high-pass filtering process. We notice that the gradient with respect to the rings variables should also be filtered.

## 4. Results   

We present here some results for both simulated and real data, and compare our method with two mainstream techniques of rings correction: sinogram pre-processing based on wavelet-Fourier de-striping (Münch *et al.*, 2009 ▸) and image correction using polar coordinates transformation (Prell *et al.*, 2009 ▸). We also present the results on simulated compressed sensing data, on which our method is well adapted on the contrary to filtered back-projection.

### 4.1. Simulated data   

We use the standard test image ‘Lena’ containing both smooth components and texture details, making it more challenging to reconstruct than usual phantoms like the Shepp–Logan phantom. For all these tests except the test on simulated compressed sensing data (Fig. 5), the image size is 512 × 512 pixels, and 800 projections were used for the reconstructions.

The tests are divided into increasing levels of difficulty for rings removal methods. In the first test, constant lines are added in the sinogram. These lines, independent of the projection angle, give rise to rings artifacts in the reconstructed slice. Since the spurious lines have a constant value, they should be well handled by pre-processing techniques.

In the second test, lines with variable intensity are added to the sinogram. This makes the correction more difficult for pre-processing techniques, especially if the lines have sharp variations (*i.e.* high-frequencies components).

In the third test, ring-shaped features are added in the phantom before adding spurious lines in the sinogram. This case is more challenging because correction methods should not remove any feature coming from the phantom (they belong to the ‘true’ image), while actually removing rings coming from the sinogram (they come from a flaw in the experimental setup).

The reference sinogram pre-processing technique is the wavelet-Fourier filtering (Münch *et al.*, 2009 ▸). This method first computes the wavelet decomposition at a level *L* of the sinogram. The vertical detail coefficients 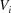 at level 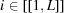 emphasize the spurious lines that give rise to rings artifacts. In these coefficients, a spurious line is nearly constant along the projection angle; thus it has only low frequencies in the Fourier domain. Filtering these few low frequencies in the Fourier domain enables the line to be suppressed after taking the inverse Fourier transform. The filter used is a high-pass Gaussian filter whose standard deviation σ tunes the bandwidth. Then, the sinogram is reconstructed from these filtered wavelet coefficients. The Matlab implementation of this method is provided in the author’s article. In the tests, σ denotes the standard deviation of the Gaussian filter and *L* is the number of levels of the wavelet decomposition.

The image correction technique used here is Rings Correction in Polar Coordinates (RCP) (Prell *et al.*, 2009 ▸). It transforms the image into polar coordinates and performs a low-pass filtering in the radial direction. The filtered image is then subtracted from the original image, and a threshold is applied to ignore non-artifact structures. The result is filtered in the azimuthal direction. After a transformation into Cartesian coordinates, the image should only contain rings artifacts; these are subtracted from the original image. A C++ implementation is given by Blair (2014 ▸). In the tests, the thresholding parameters are set so that all the image pixels can be considered as possibly part of an artifact. The important remaining parameters are the maximum ring width *W*, and the maximum angular arc 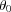 we expect the rings to have.

**Figure 2:**
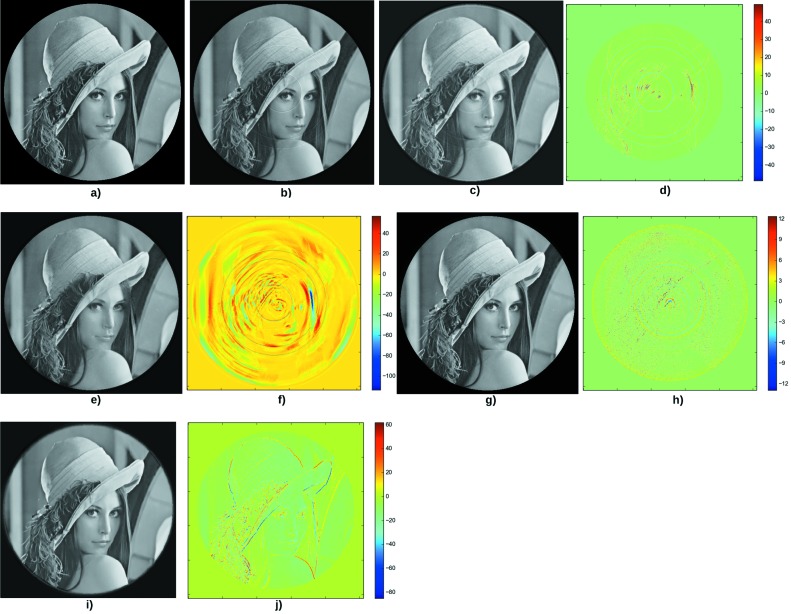
First test case. (*a*) Original phantom. (*b*) Result of filtered back-projection after adding constant lines in the sinogram. (*c*) Image back-projected after applying the Munch *et al.* de-striper algorithm with 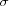 = 3.5, 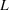 = 2 and the ‘Daubechies 15’ wavelet. (*d*) Difference between the phantom and the corrected image. The PSNR is 26.6. (*e*) Result of the correction with the RCP technique with 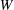 = 10 and 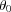 = 10. (*f*) Difference between the phantom and the corrected image. The PSNR is 29.6. (*g*) Result of the reconstruction using the Total Variation regularization, with parameters 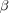 = 0.5, 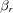 = 0.05. (*h*) Difference between the phantom and the corrected image. The PSNR is 39.0. (*i*) Result of the reconstruction using the Dictionary Learning technique with 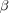 = 0.3, 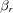 = 0.5, 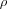 = 1. (*j*) Difference between the phantom and the reconstructed image. The PSNR is 29.2.

Fig. 2 ▸ shows the results for the first test case. The rings are reduced by the sinogram pre-processing technique (Fig. 2*c*), but they do not totally disappear. Besides, additional artifacts appear after the correction. The RCP performs slightly better (Fig. 2*e*); a strong artifact is added to the right but the result is qualitatively better. The Total Variation regularization entirely removes the rings (Fig. 2*g*). It can be seen (Fig. 2*h*) that other rings were actually added to the slice, but their amplitude is very small according to the scale (color bar), so they are not detrimental to the reconstruction quality. The Dictionary Learning reconstruction (Fig. 2*i*) removes the rings, but the difference image (Fig. 2*j*) shows that the slice is slightly blurred: the very fine details are smoothed.

**Figure 3:**
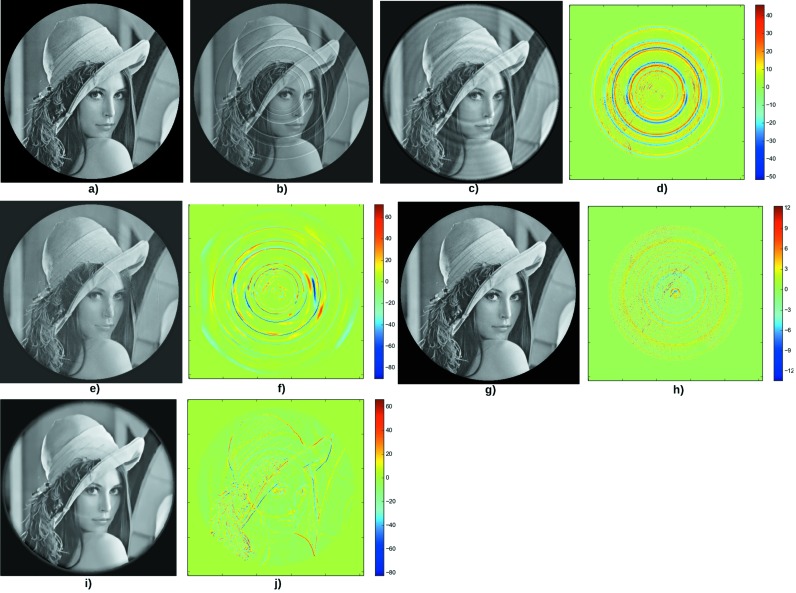
Second test case.(*a*) Original phantom. (*b*) Result of filtered back-projection after adding lines of variable width and intensity in the sinogram. (*c*) Image back-projected after applying the Munch *et al.* de-striper algorithm, with 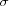 = 1.5, 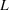 = 2 and the ‘Daubechies 15’ wavelet. (*d*) Difference between the phantom and the corrected image. The PSNR is 29.2. (*e*) Result of the correction using the RCP technique with 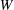 = 10 and 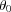 = 10. (*f*) Difference between the phantom and the corrected image. The PSNR is 25.1. (*g*) Result of the reconstruction using the Total Variation regularization, with parameters 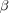 = 0.5, 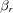 = 0.05. (*h*) Difference between the phantom and the corrected image. The PSNR is 39.4. (*i*) Result of the reconstruction using the Dictionary Learning technique with 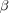 = 0.7, 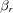 = 0.5, 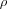 = 1. (*j*) Difference between the phantom and the reconstructed image. The PSNR is 30.6.

Fig. 3 ▸ shows the results for the second test case. The sinogram filtering adds many spurious rings (Fig. 3*c*). The RCP technique removes most of the rings, but small rings details remain. The difference image (Fig. 3*f*) shows some artifacts which may be the result of the different transformations between Cartesian and polar coordinates. The Total Variation regularization (Fig. 3*g*) entirely removes the rings artifacts; the result is qualitatively very close to the original phantom. On the other hand, the Dictionary Learning reconstruction does not manage to perfectly correct the slice: a remaining ring can be seen on Lena’s cheek (Figs. 3*i* and 3*j*). A solution can be to increase the value of the parameter β [*i.e.* 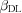 in equation (10)], but it would lead to a more blurry image. This suggests that the Total Variation is more suited than Dictionary Learning to correct the rings on a image containing many texture components.

**Figure 4:**
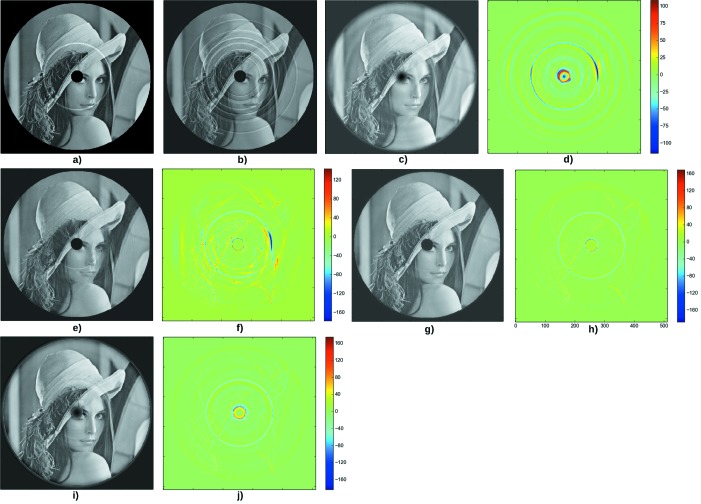
Third test case. (*a*) Original phantom. (*b*) Result of filtered back-projection after adding lines of variable width and intensity in the sinogram. (*c*) Image back-projected after applying the Munch *et al.* de-striper algorithm, with 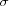 = 2.5, 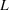 = 5 and the ‘Daubechies 20’ wavelet. (*d*) Difference between the phantom and the corrected image. The PSNR is 23.2. (*e*) Result of the correction using the RCP technique, with 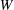 = 10 and 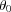 = 10. (*f*) Difference between the phantom and the corrected image. The PSNR is 21.2. (*g*) Result of the reconstruction using the Total Variation regularization, with parameters 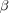 = 0.5, 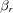 = 0.05. (*h*) Difference between the phantom and the corrected image. The PSNR is 29.9. (*i*) Result of the reconstruction using the Dictionary Learning technique with parameters 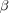 = 0.05, 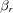 = 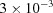, 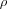 = 10. (*j*) Difference between the reconstruction and the phantom. The PSNR is 28.3.

Fig. 4 ▸ shows the results for the third test case. Here two features are added to the original phantom (Fig. 4*a*): a black disk and a circular ‘ring’. These features are part of the phantom; they should not be filtered by rings correction techniques. Lines added to the sinogram are not constant along the projection angle, and their width can be several pixels. This leads to a back-projected image (Fig. 4*b*) with large rings. The RCP technique (Fig. 4*e*) is more efficient than the sinogram pre-processing (Fig. 4*c*).

The Total Variation (Fig. 4*g*) removes the rings, but also nearly removes the circular feature of the phantom (Fig. 4*a*), which gives the blue circle in the difference image (Fig. 4*h*). The black disk is well preserved.

The Dictionary Learning technique (Fig. 4*i*) does not give as good results as the Total Variation regularization. The black disk is blurred, and the rings are not entirely corrected.

**Figure 5:**
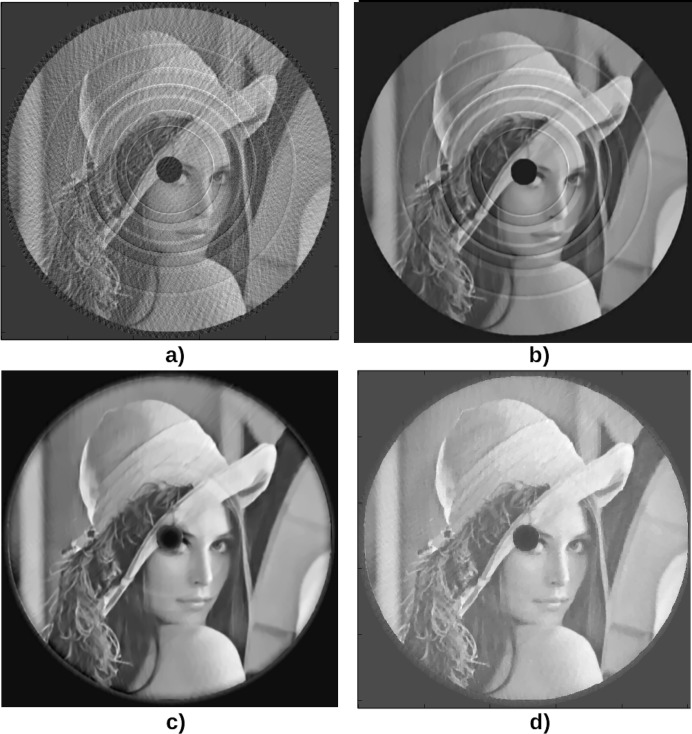
Reconstruction of the third case phantom (Fig. 4*a* ▸) with 80 projections instead of 800. (*a*) Result of the reconstruction using the filtered back-projection. (*b*) Result of the reconstruction using the Dictionary Learning technique, without rings correction, with parameters 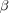 = 0.05, 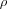 = 10. (*c*) Result of the reconstruction using the Dictionary Learning with rings correction, with parameters 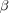 = 0.05, 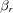 = 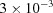, 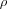 = 10. (*d*) Result of the reconstruction using the Total Variation regularization with parameters 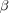 = 1, 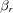 = 0.05.

In practice, compressed sensing is especially interesting when it comes to reconstructing a volume from few projections. In the previous test cases, the 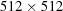 image needed 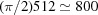 projections to be accurately reconstructed with the filtered back-projection. Fig. 5 ▸ shows the result of the third test case with 80 projections instead of 800. Filtered back-projection (Fig. 5*a* ▸) leads to star artifacts due to the data undersampling. The reconstruction is much more satisfactory with Dictionary Learning or TV regularization. In these iterative methods, the ring artifacts correction can be turned off (Fig. 5*b* ▸) or on by simply adjusting one parameter. In all cases, the black disk is preserved and the ring artifacts correction did not suffer from the small number of projections. One can notice that, in this case, DL produces smoother results than TV, but does not entirely remove the rings (Fig. 5*c* ▸) while TV is able to entirely remove the rings (Fig. 5*d* ▸).

**Figure 6:**
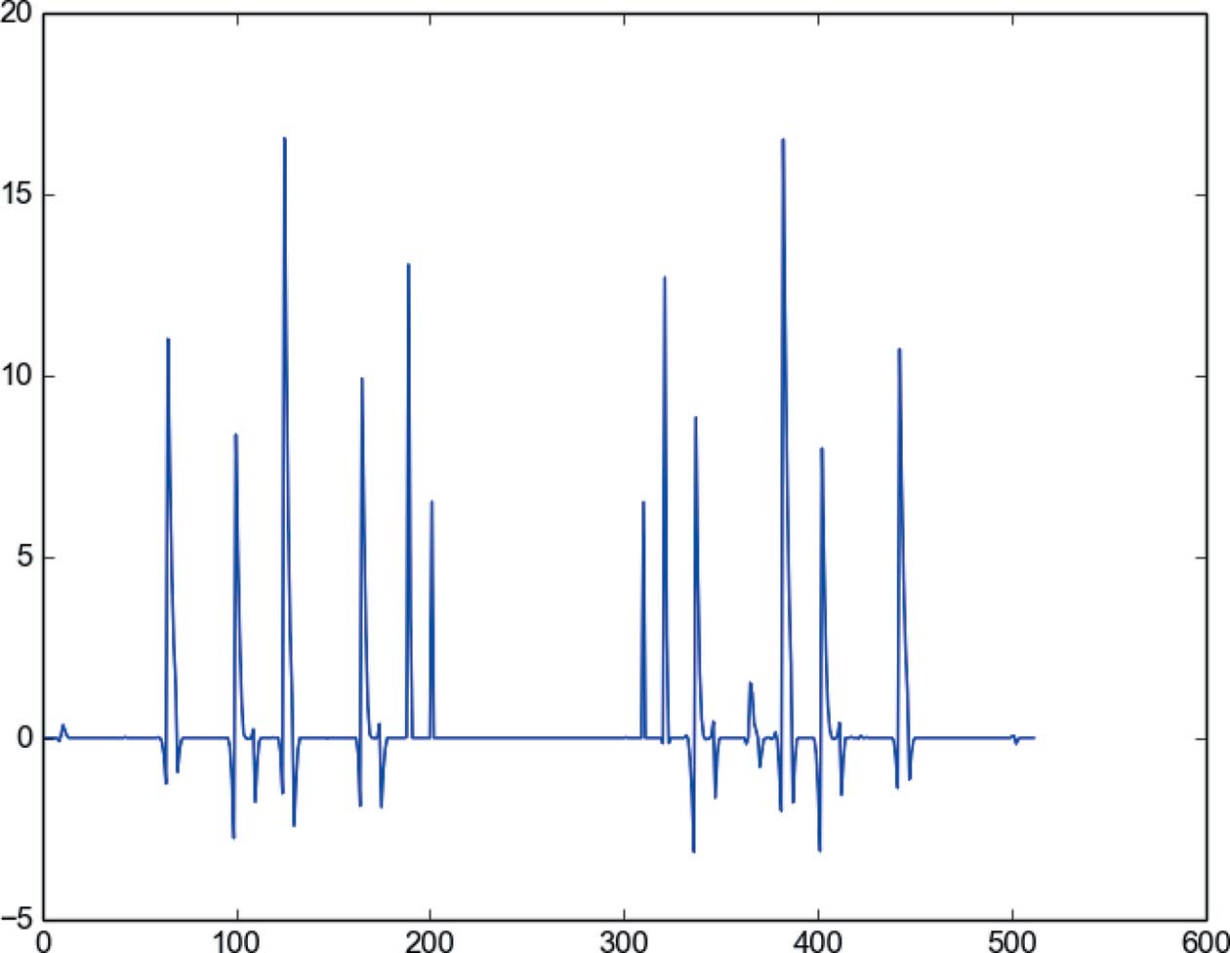
Vector of rings variables for the second test case (Fig. 3*b* ▸). The horizontal axis goes from zero to the number of bins of the detector; that is, in this simulated case, 512 for the 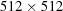 test image.

A plot of the rings variables during the Total Variation reconstruction shows that the rings are actually captured by the ring vector 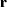. The six peaks representing the detected lines in the sinogram are clearly visible in Fig. 6 ▸.

Beside the visual aspect of the corrected image, we use the peak signal to noise ratio (PSNR) as a measure of the correction quality. Although PSNR gives a score of the overall similarity between the corrected image and the original phantom, it is inconsistent with the eye perception of quality. For example, RCP performed better than sinogram filtering in these tests, but had the lowest PSNR for cases 2 and 3. The structural similarity index gives the same kind of results. The reasons for this inconsistence can be the following. The Munch filter has a blurring effect, since it modifies the wavelet detail coefficients, which is detrimental to the overall image quality. However, the blurring effect is averaged in the MSE/SSIM (mean squared error/structural similarity) calculation. On the other hand, the polar filter does less blurring, but there are strong local errors, leading to a high MSE. Quantitative quality assessment is a difficult issue in tomographic reconstruction, and to our knowledge no satisfactory metric adapted to tomographic reconstruction has been proposed yet.

For these tests, the sinogram pre-processing technique did not yield good results. This can be due to the fact that the sinogram lines were captured by the wavelet approximation coefficients rather than by the detail coefficients, making the filtering ineffective. By trying with lines of smallest amplitude, the wavelet-Fourier method actually worked without adding large artifacts in the reconstructed image.

### 4.2. Experimental data   

We give here some results for reconstructions performed on real data. The samples were kindly provided by the ESRF beamline ID19.

#### 4.2.1. Syntactic foam   

**Figure 7:**
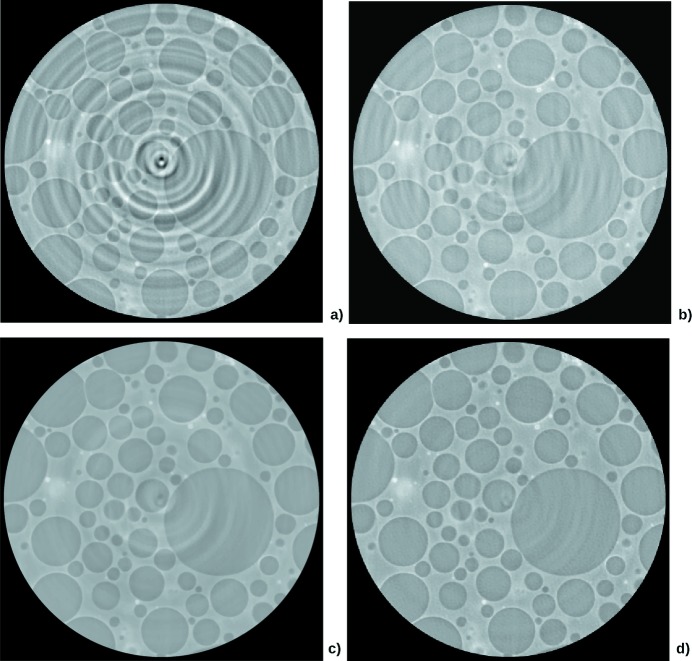
Syntactic foam sample tomography acquired at ESRF ID19, with energy of 19 keV and pixel size of 0.28 µm. (*a*) Filtered back-projection. (*b*) Correction with RCP technique, using the parameters 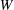 = 60 and 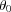 = 60. (*c*) Reconstruction with Total Variation technique, using the parameters 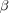 = 0.35 and 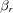 = 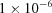. (*d*) Reconstruction with Dictionary Learning technique, using the parameters 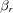 = 0.1, 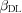 = 0.035 and 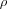 = 20.

The reconstruction technique was used on a syntactic foam sample. The slice was 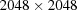 pixels, and 2449 projections were used. In this case, the rings are ‘large’ to the extent that the radius difference between the exterior and the interior of the ring is several pixels. This means that the spurious lines in the sinogram have several pixels of width along the detector bins axis, forming ‘bands’. However, the intensities of the lines forming a band has too many variations to be efficiently filtered by sinogram pre-processing techniques. This case is also difficult for slice-correction algorithms which detect circular features, since the sample itself has circular features which should not be removed. The RCP technique (Fig. 7*b* ▸), however, only detects circular features whose center is the image center. The Total Variation technique (Fig. 7*c* ▸) removes most of the rings, but a relatively high β had to be chosen, which led to a somewhat blurred result. The Dictionary Learning technique (Fig. 7*d* ▸) performs a better correction. Note that the dictionary has been learned offline on the Lena image, and yet provided a satisfactory reconstruction.

#### 4.2.2. Rhynie chert   

We applied the reconstruction on a rhynie chert sample. The slice was 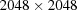 pixels, and 2000 projections were used. This situation is almost the opposite of the previous case: the rings artifacts have a small intensity in the reconstructed slice, and the sample borders form a nearly circular polygonal shape. These borders have a huge amplitude with respect to the rest of the sample, and the transition between the border and the interior/exterior is very sharp. Thus, slice correction techniques would try to remove the borders before any other feature in the slice depending on the thresholding parameters.

**Figure 8:**
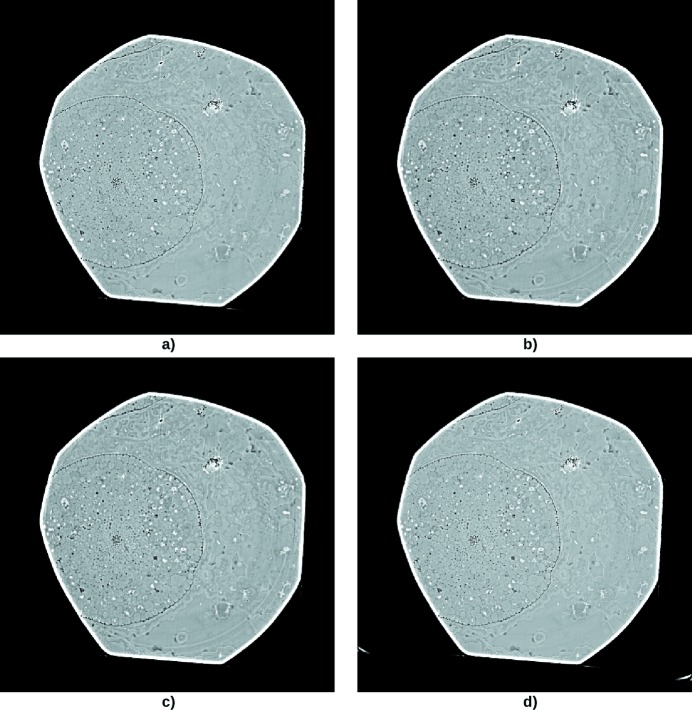
Rhynie chert sample tomography acquired at ESRF ID19, with energy of 17.6 keV and pixel size of 1.52 µm. (*a*) Filtered back-projection with the correct rotation axis. (*b*) Correction with the RCP technique, using the parameters 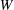 = 10 and 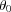 = 10. (*c*) Reconstruction with the Total Variation regularization using the incorrect rotation axis. (*d*) Reconstruction with the Total Variation regularization using the correct rotation axis. The parameters were 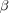 = 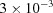 and 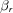 = 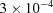.

We realised that the rings correction was difficult for the Total Variation reconstruction: the procedure added rings tangent to one of the slice borders. It turned out that the problem was due to the rotation center for (back)projection being improperly set, leading to accumulating errors in the iterative reconstruction. Indeed, Total Variation and Dictionary Learning reconstruction require to compute the projection for the functional, and the back-projection for the functional gradient. If the rotation center for these operations is not the same as the one used for actually rotating the sample, slight errors appear in the (back)projection; these errors accumulate with the number of iterations and take the form of circular features (Fig. 8*c* ▸).

After setting the correct rotation center, we were able to remove the ring artifacts (Fig. 8*d* ▸), especially the one near the center of Fig. 8(*a*) ▸. In this case, the RCP technique performed quite well (Fig. 8*b* ▸).

### 4.3. Execution time and convergence rate   

In this section we measure the execution time required to obtain an acceptable reconstruction. All the tests are performed on a machine with an Intel Xeon CPU E5-1607 v2 @ 3.00 GHz processor and a GeForce GTX 750 Ti graphic card.

**Figure 9:**
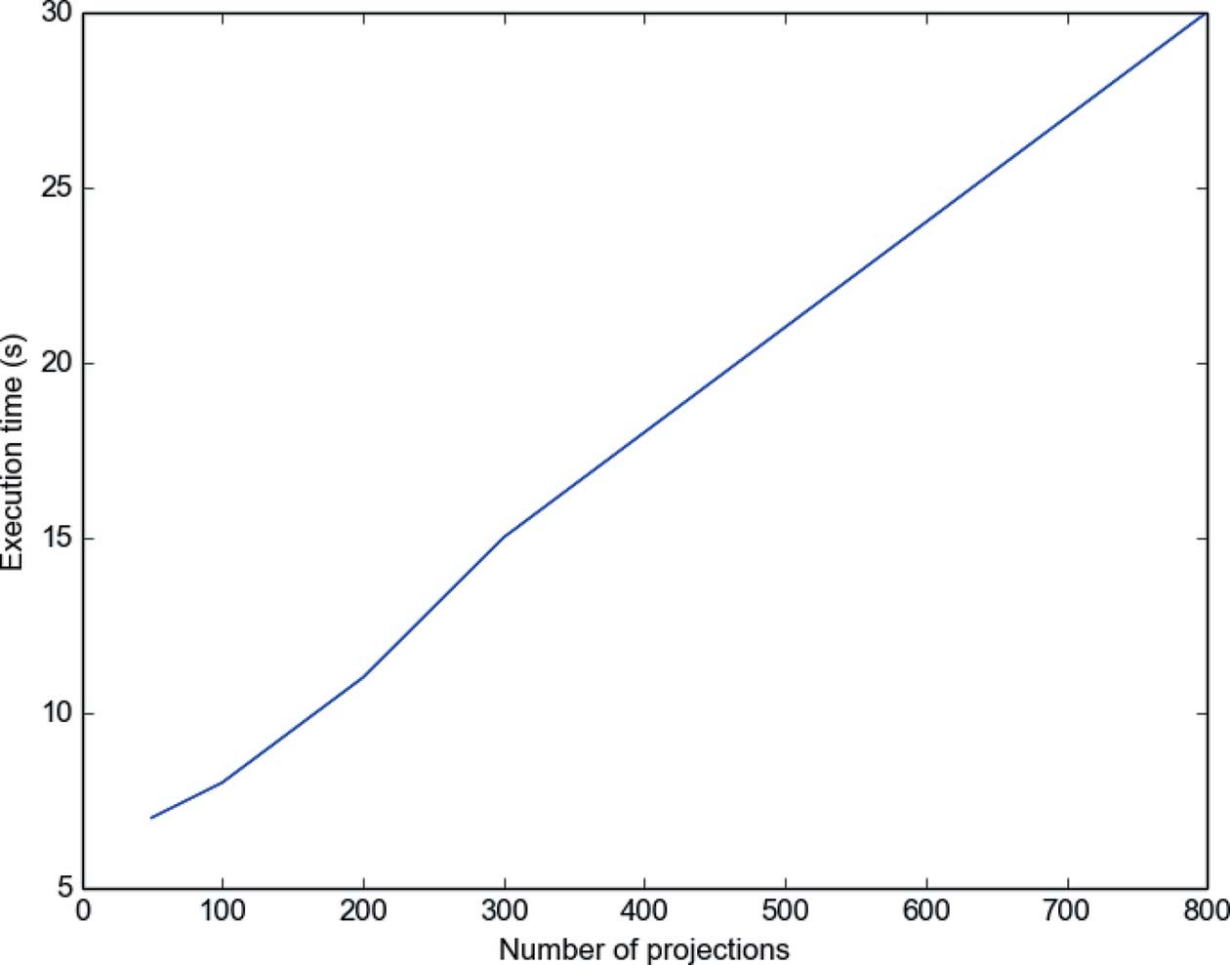
Execution time as a function of the number of projections for 1000 iterations. The image used is the 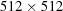 test image ‘Lena’ corrupted with the rings presented in the second test case.

We measured that the execution time is the same with rings correction and without rings correction: including the ring artifacts correction in the functional has no additional cost in the reconstruction. The execution time is proportional to the number of projections, as can be guessed with Fig. 9 ▸, since more data have to be processed by the operators.

The values of the objective function as a function of the number of iterations is an illustration of the convergence rate. For Total Variation reconstruction, the objective function is given by equation (12); it includes both the fidelity term (Euclidean distance) and the regularization term (L1 norm of the image gradient). For Dictionary Learning, it is given by equation (17).

**Figure 10:**
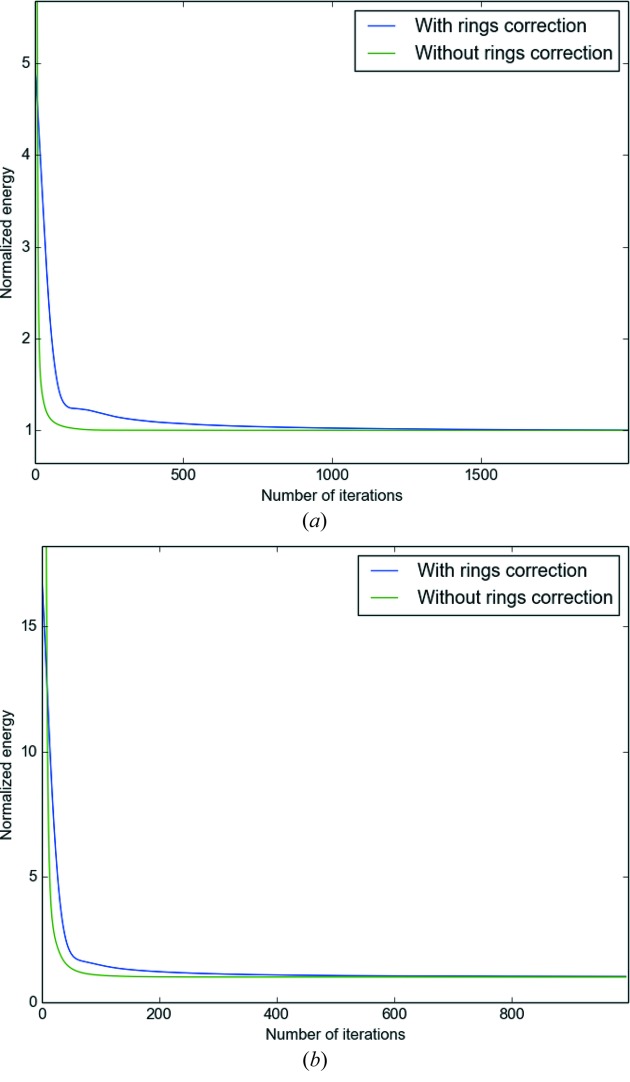
Energy as a function of the number of iterations for the Total Variation tomographic reconstruction. The energy is normalized by the energy of the last iteration in order to have the same scale in the two cases. The image used is the 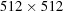 test image ‘Lena’ corrupted with the rings presented in the second test case. (*a*) Evolution of energy with 800 projections. (*b*) Evolution of energy with 200 projections.

Fig. 10 ▸ shows the evolution of the objective function [equation (12)] as a function of the number of iterations. With rings correction, the reconstruction process needs more iterations to converge. For simulated and real data, it turned out that a satisfactory reconstruction can be achieved with less than 1000 iterations without rings correction. When the rings correction is activated, it takes about 2000 iteration to correctly remove the ring artifacts.

Thus, while the rings correction has no additional cost per iteration, it takes nevertheless more iterations to converge to an image with removed ring artifacts. The ‘energy transfer’ between the fidelity term 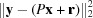 and the L1 norm of the rings 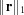 is actually quite slow. Using another optimization algorithm might accelerate the convergence of the joint optimization with respect to 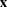 and 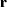.

The convergence rate also slightly depends on the number of projections. Fig. 10 ▸ shows that the reconstruction process converges in 500 iterations (400 with rings correction) for 200 projections, when it takes about 2000 iterations (1500 without rings correction) for 800 projections. This is due to the fact that the weight of the fidelity term virtually increases as the number of projections increases. To counter-weight this, one has to increase the penalty term β of the regularization, which makes the energy transfer between the fidelity term and the ring variables a little faster.

The reconstruction parameters like the Total Variation penalization and rings correction weight depend on the data. For most data in parallel geometry, the same parameters can be used for all the slices. Thus, one single slice can be used for the parameters optimization. The parameters are chosen manually to have a good reconstruction quality. An approach towards automatic parameters optimization might be the L-curve method (Hansen & O’Leary, 1993 ▸), which is not used here.

## 5. Conclusions   

We have presented a new way to correct the rings artifacts that appear in tomographic reconstruction. This technique fits well in the scope of compressed sensing tomographic reconstruction, since it is especially adapted when the number of projections is limited. Including the rings artifacts correction in the iterative reconstruction process has shown to be efficient while requiring no extra pre- or post-processing steps. Besides, additional artifacts are less likely to appear thanks to the regularization. This method can be adapted to any compressed sensing approach, since the only things to do are modifying the functional and the iterative correction step accordingly.

In a further work, we would like to improve the convergence rate of the rings correction in order to make the method more attractive, for example using other optimization algorithms. We also would like to extend this method to lines that are not constant along the projection angle in the sinogram, in order to cover more general and difficult cases.
